# Fruit Syrups

**Published:** 1870-08

**Authors:** 


### FRUIT SYRUPS.

Almost all the colored fruit syrups contain arsenic; a few drops of aquafortis brightens real fruit colored syrups and gives a yellowish tinge to poisonous colors. The use of syrups diluted with water as a drink in summer is not philosophical, just because sweets beget thirst and they are naturally heating. For all ordinary purposes cold water without ice is the safest and most healthful.

### HYGIENE IN SCHOOLS.

Thirty-five years ago we began to write about the importance of having the general principles of health taught in public schools, especially in theological seminaries. But our arguments must have been as weak as water, for they failed to astonish the world or even to attract public attention, but the French at Montfort have made it a branch of primary school study. If these teachings were confined to impressive illustrations of the importance of

Regularity in eating,
Abundant sleep,
Attention to the bodily functions, an amount of disease would be prevented in a few years beyond any ordinary conception. The foundation of half the common diseases which embitter later life are laid before the first score of years is completed.

